# Epigenetic regulation in human cancer: the potential role of epi-drug in cancer therapy

**DOI:** 10.1186/s12943-020-01197-3

**Published:** 2020-04-27

**Authors:** Yuanjun Lu, Yau-Tuen Chan, Hor-Yue Tan, Sha Li, Ning Wang, Yibin Feng

**Affiliations:** School of Chinese Medicine, The University of Hong Kong, 10 Sassoon Road, Pofulam, 000000 Hong Kong, Special Administrative Region of China

**Keywords:** Epigenetic modification, Cancer, Epi-drugs, Combination strategy, Tumour microenvironment

## Abstract

Epigenetics is dynamic and heritable modifications to the genome that occur independently of DNA sequence. It requires interactions cohesively with various enzymes and other molecular components. Aberrant epigenetic alterations can lead to inappropriate onset of genetic expressions and promote tumorigenesis. As the epigenetic modifiers are susceptible to extrinsic factors and reversible, they are becoming promising targets in multiple cancer therapies. Recently, various epi-drugs have been developed and implicated in clinical use. The use of epi-drugs alone, or in combination with chemotherapy or immunotherapy, has shown compelling outcomes, including augmentation of anti-tumoral effects, overcoming drug resistance, and activation of host immune response.

## Introduction

The term “epigenetics” was addressed initially as “the branch of biology which studies the causal interactions between genes and their products, which bring the phenotype into being” by C.H. Waddington in the 1940s [1]. From then on, implications of epigenetics have been extended to a wide range of biological processes over time as accumulating evidence suggested that heritable changes to the genome occur independently of alterations in somatic cells regardless of their differentiation status [2]. The heritable changes, either occurring or maintaining during multiple cellular biological processes with the same genetic information, require fine-tuned epigenetic modifications, which commonly including DNA methylation, histone, or chromatin post-translational modifications (PTM), as well as non-coding RNAs regulations. Failure of heritability of epigenetic marks may result in inappropriate initiation or inhibition of gene expressions and lead to pathological changes, including cancers [3, 4].

Cancer is a consequence of accumulative genetic mutations in concert with epigenetic alterations, as well as environmental factors. A large number of studies have been taking great efforts in characterizing the genomic landscape of cancers from oncogene-driven signalling pathways to the mutation spectrum in different cancer subtypes. Distinct from genetic mutation, epigenetic influences refer to modifying gene expression without permanent changes in the genomic sequence. They are preferentially applied in cancer cells given that epigenetic alterations are reversible and faster regulated compared to genomic evolution [5]. Except for the fundamental changes that occur to the somatic cells, other multiple forces are cohesively shaping the landscape of cancer, thus bringing into additional dimensional complexity. The tumour microenvironment (TME) consists of supporting texture and cells and establishes a niche to fuel tumour cells with a multitude of stromal factors. Current epigenetic modifications are not only focused on the progress of cancer cells development, but also the tumour cells-TME interactions.

Given the importance of epigenetic regulation in cancers, the treatment targeting epigenetics is becoming an attractive strategy of cancer therapy. Epigenetic treatment may therefore benefit cancer patients as monotherapy and a combinatory treatment with other current therapy. In this review, we summarize the mechanisms of epigenetic modifications in tumorigenesis, and we also envision more advanced sequencing technologies that would be available for epigenome mapping and enable epigenetic modifications precisely applied in cancer therapy. The drawback and potential pitfall of current epigenetic drugs are also discussed. We hope our review could shed light on the significance of epigenetics in the development and treatment of cancer.

## Mechanisms of epigenetic modifications

The epigenetic modifications can be generally categorized into three groups: DNA and RNA methylations, histone modifications, and non-coding RNAs, which are considered as main mechanisms of regulation during carcinogenesis/cancer progression.

### DNA and RNA methylations

#### DNA methylation and demethylation

DNA methylation is the most extensively studied epigenetic mechanism that predominantly occurs in CpG islands (CGIs) where preferentially located at the 5′ promoter region of more than 50% of human genes [6, 7]. It displays a fundamental function in development and diseases, including X chromosome inactivation, embryonic development, genomic imprinting, epigenetic reprogramming, cell identity establishment, and lineage specification [8–10]. Generally, it exhibits gene silencing via covalent addition of methyl groups from S-adenosylmethionine (SAM) to the 5 position of the cytosine pyrimidine ring. The 5-methylcytosine (m5C) structure can either prevent access of transcriptional factors (TFs) to the binding sites of DNA, or recruit methyl-binding domain proteins (MBDs) in association with histone modifications to reconfigure chromatin, thus leading to repressive gene expression.

Three DNA methyltransferases (DNMTs), namely DNMT1, DNMT3a, and DNMT3b, are orchestrated in catalysing DNA methylation. DNMT1, the maintenance DNA methyltransferase, has a higher catalytic activity to preferentially methylate hemimethylated DNA during replication and is mostly responsible for maintaining the DNA methylation status [11, 12]. While the precise DNA methylation status in the genome is generated and supported by “de novo” methyltransferases, DNMT3a and DNMT3b, they display equal preference to bind to the previously unmethylated DNA independently of replication [13].

In contrast, DNA demethylation is a reverse action that recovers silenced genes affected by DNMTs. It is catalysed by a family of Ten-eleven translocation methylcytosine dioxygenases (e.g., TET1, TET2, and TET3), which can turn 5mC to 5-hydroxymethylcytosine (5-hmC), even further oxidize 5-hmC into 5-formylcytosine (5-fC) and 5-carboxylcytosine (5-caC) [14, 15]. Homeostasis between the demethylation and methylation of the genome incurs as a dynamic mechanism of gene expression in various types of cells.

#### RNA methylation

N^6^-methyladenosine (m^6^A), referring to the methylation of adenosine residue at the N-6 position, was first discovered in the 1970s and is emerging as a hotspot issue in epigenetic mechanisms, as well as in cancer biology. M^6^A modification enriches near the stop codon, 3’UTR, and within internal long exons [16, 17]. It affects almost every aspect of RNA processing, including RNA transcription, degradation, splicing, and translation [18].

Recent studies have found that m^6^A modifications are reversible and dynamic. RNA m^6^A formation requires multiple methyltransferase components categorized as “writers”, including methyltransferase-like 3 (METTL3), METTL14, METTL16, Wilms tumour 1-associated protein (WTAP), RNA binding motif protein (RMB15/15B), zinc finger CCCH-type containing 13 (ZC3H13), and KIAA1429 [19–23]. While decoding of m^6^A methylation can be achieved by interactions among components of “erasers” (e.g. FTO and ALKBH5) and “readers”, such as YT521-B homology (YTH) domain-containing proteins, eukaryotic initiation factor 3 (eIF3), heterogenous nuclear ribonucleoprotein (HNRNP) protein family, and insulin-like growth factor 2 mRNA binding proteins (IGF2BP) family [24–27].

### Histone modifications

In chromatin, DNA is packaged into a highly compact structure wrapped with histone octamer, thereby forming nucleosomes and the so-called “beads on a string” structure, which facilitate controlling the accessibility of DNA sequence. Each histone octamer is composed of a tetramer of two copies of histone 2A (H2A) and two copies of histone 2B (H2B), flanked by dimers of histone 3 (H3) and histone 4 (H4). These histone proteins contain a globular C-terminal domain and an extended N-terminal tail, which are subject to various PTMs, including methylation, acetylation, ubiquitylation, phosphorylation, SUMOylation, ADP ribosylation, citrullination, and biotinylation at specific amino acidic residues.

Among those PTMs, acetylation, and methylation of lysine residues on H3 and H4 have been mostly studied. The mechanism of histone acetylation is based on the “charge neutralization model” that the positive charge of lysine residues on H3/H4 facilitates a tight packaging of negatively charged DNA with histones. Whereas the addition of an acetyl group can lose up the tight configuration of chromatin, thus enabling transcriptional factors (TFs) access for transcription [28]. Multiple enzymes are responsible for catalysing the addition and removal of acetyl groups, including histone acetyltransferases (HATs) and histone deacetylases (HDACs) respectively.

Unlike histone acetylation, the effect of histone methylation is more complicated and dependent on the targeted residues. For example, methylation at lysine 4/36/79 of histone H3 (H3K4/36/79) typically contributes to active transcriptional status, while methylation at H3K9/27 and H4K20 is generally considered to be repressive epigenetic marks [29–32]. They are exclusively catalysed by different histone methyltransferases (HMTs) that most of them contain a SET domain. For example, enhancer of zeste 2 (EZH2) is specific for H3K27 trimethylation (H3K27me3) that exerts transcriptional silencing function [33]. SET7/9 catalyses H3K4me that activates the expression of inflammatory genes [34]. Reversely, removing methyl groups from those marks by histone demethylases (HDMTs) also alters the status of transcriptional activity.

### Non-coding RNAs

Non-coding RNAs (ncRNAs) take up more than 70% of human genome and have regulatory effects [35]. They are mainly categorized into small ncRNAs (sncRNAs, < 200 nt) and long ncRNAs (lncRNAs, > 200 nt) based on size. The most characterized sncRNA is miRNA, a highly conserved single-stranded RNA with ~ 20 nucleotides. They were considered as “junk transcripts” upon their discovery; however they are critical mediators in biological robustness by buffering off the small perturbations, thus ensuring homeostasis of organisms. Almost 60% of protein-coding genes are subject to miRNAs regulation in humans [36]. They downregulate gene expression via complementarily binding to the 3′ UTR of target mRNA. The role of miRNAs, either onco-miRNAs or tumour suppressors, depends on the role of their target genes. More than 50% of miRNA genes are located closely within CGIs, thereby they are susceptible to other epigenetic modifications [37]. Nowadays, a growing number of studies have revealed the mechanisms of miRNAs in almost all types of cancers.

LncRNAs represent a diverse family of long transcripts generated from different genomic locations. They can influence the target sites either within the nucleus or cytoplasm and play many characters such as chromatin regulators, enhancers, ncRNAs sponge, molecular scaffold, etc. In general regards, lncRNAs are similar with mRNAs that undergo splicing, 5′-cap, and polyadenylation, except newly discovered circular RNAs (circRNAs) which have no cap and poly-A tail [38]. Recent advances have rectified that lncRNAs and circRNAs enable to encode functional peptides with short open reading frames (sORFs), which make their mechanisms more complicated [39].

## Aberrant epigenetic changes in cancer development

### Intratumor epigenetic alterations

Tumorigenesis is a consequence of accumulating changes in genome and epigenome, which confers tumours with traits of heterogeneity and plasticity. Two models have been proposed to decipher how tumours progress: (i) the cancer stem cell (CSC) model, also termed the hierarchical model, regards cancer stem cells as the origin of oncogenic transformation; (ii) the stochastic or clonal evolution model suggests that the initial oncogenic change is acquired progressively by non CSCs [40, 41]. There is no doubt that epigenetic alterations are critically involved in both models as they can regulate genetic deregulation and certain mutations.

**Fig. 1:**
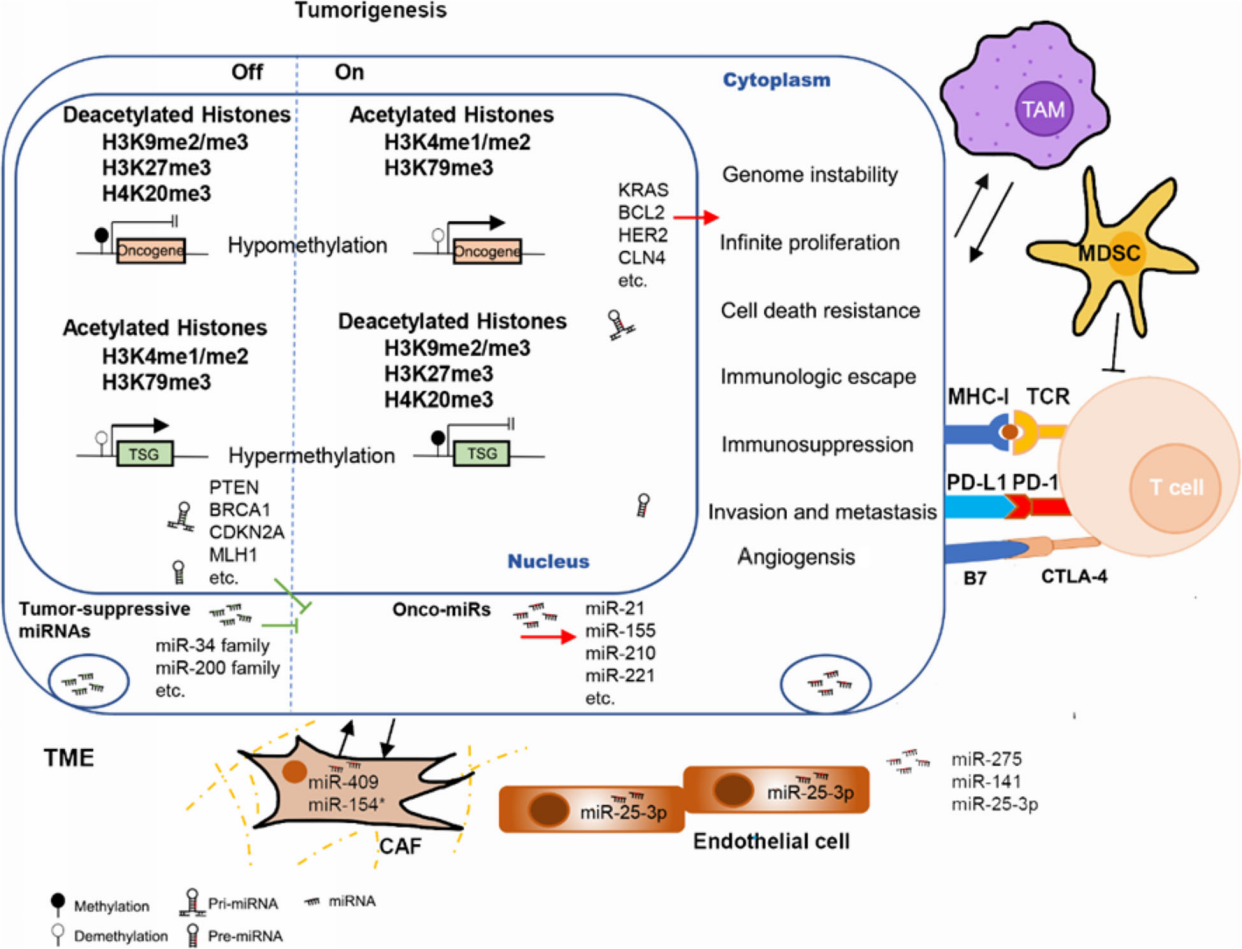
Aberrant epigenetic alterations in tumour development. Within tumour cells, the activated epigenetic modifiers switch on the transcription of oncogenes and onco-miRNAs, assisting the formation of cancer hallmarks. However, the suppressive epigenetic modifiers switch off the transcription of TSGs and tumour-suppressive miRNAs, which exert inhibitory effects on tumorigenesis. The TME is subject to epigenetic modifications and assists in creating a pre-metastatic and immune suppressive niche for tumour cells

The activation of oncogenes and/or suppression of tumour suppressor genes (TSGs) are considered to be one of factors contributed to the onset of cancer, and they are always consistent with epigenetic changes. DNA methylation acts as a switch controlling the “open” and “off” status of the gene expression (Fig. 1). Hypermethylation of promoters in CGIs is the most recognised mechanism of epigenetic alterations in cancer cells and has been well implicated in various cancer types. Abundant TSGs are under hypermethylation, such as RASSF10 in kidney cancer [42], SIX3 in glioblastoma [43], CDKN2A and PTEN in melanoma [44, 45], etc. Besides, not only TSGs hypermethylation is commonly seen in many cancers, but additional genes involved in multiple pivotal cellular functions also present hypermethylation. The studies of prostate cancer (PC) have demonstrated that the heavily methylated situation occurred on the promoters of glutathione S-transferase pi (GSTP1) and other genes such as CDKN2A, TIMPS, and DAPK, which participate in cell cycle, cell metastasis, apoptosis and so forth [46]. By contrast, hypomethylation of oncogenes is commonly reported in multiple cancers, including LY6K in glioblastoma [47], SLC34A2 in papillary thyroid carcinoma [48], RBBP6 in colorectal cancer [49], etc. Moreover, instead of targeting specific genes individually, the variation of DNA methylation is likely to be a genome-wide regulatory scheme. Latest study of genomic and epigenomic landscape in hepatocellular carcinoma (HCC) found that hyper- and hypomethylation occurred in the early preneoplastic phases of HCC, which was significantly relevant to deregulation of cancer-related genes [50].

Apart from aberrant DNA methylation, unbalanced histone modification is abused by those cancers that adhere to the CSC model, in which bidirectional interconversions are essential. The bivalent histone marks, activating H3K4me3 and repressive H3K27me3, are originally addressed in the differentiation of embryonic stem cells (ESCs) [51]. However, some types of cancers partially recapitulate this bivalency to deregulate oncofetal genes in cancer cells [52–54]. In colorectal cancer, some genes that supposed to be stem cell markers underwent H3K27me3 loss, such as SOX9, LGR5, ASCL2, OLFM4, EPHB3 [55]. In addition, the bivalency of histone marks on EMT-related genes (e.g. CDH1, SNAI1, TWIST1) confers them bipotential capacity to potentiate cancer plasticity [56–58].

Aberrant epigenetic alterations on chromosome are dimensionally complex as they are likely to coact during tumorigenesis. Recent studies have demonstrated that histone modifications can be altered along with abnormal DNA methylation or noncoding RNAs. Even one type of histone alteration can affect other histone marks. For example, H3K36me2 expansion caused by NSD2 overexpression in multiple myeloma favored the enrichment of H3K27ac and interactions with other regulatory elements, thus activating oncogenic pathways [59]. Increasing evidence supported that abnormal epigenetic changes may either arise stochastically or be driven by transcriptional program, indicating that the mutations in key elements of epigenetic regulation (e.g. DNMTs, TETs, EZH2, etc.) or specific signalling pathways (e.g. EGFR and KRAS signalling) can modify epigenome [43].

In addition to abnormalities at chromosome level, variations in ncRNAs and RNA modifications are very common in cancer cells. Up to now, tons of aberrant miRNAs are being found in almost every type of cancers. The putative onco-miRNAs (e.g. miR-21, miR-155, miR-210, miR-221, etc.) are usually upregulated in cancer and confer cancer cells advantageous traits by targeting TSGs. By contrast, tumour suppressor miRNAs (e.g. miR-34 family, miR-200 family, etc.) exhibit the opposite function in cancers. Of note, some miRNAs serve dual roles, even in a type of cancer. For example, miR-181 family consists of four members miR-181a to miR-181d that exhibit inconsistent expression in many solid cancers, indicating they might be onco-miRNAs as well as tumour suppressor miRNAs [60]. It is intriguing that those members of miR-181 family are located at different clusters of chromosomes and subject to different epigenetic modifications, suggesting that miRNA biogenesis can be modified by other epigenetic modifications. Moreover, the interactions among different epigenetic mechanisms can either synergistically or antagonistically alter genetic expression. Another example is that the miR-200 family can target ZEB1 and ZEB2 and inhibit EMT-related genes transcription [61–63]. However, these miRNAs are subject to methylation by DNMT together with the trimethylation of H3K27 and lose control to the EMT phenotype [64–66]. Except the epigenetic effects on miRNAs biogenesis, mature miRNAs can lose their functions by lncRNAs and circRNAs. It has been widely reported that lncRNAs and circRNAs act as miRNA sponge via directly binding to the miRNAs, thus partially abrogating their roles [67]. A latest study in triple-negative breast cancer (TNBC) identified the lncRNA FAM83H-AS1 sequestered miR-136-5p which supposed to inhibit MTDH-induced proliferation, migration, and invasion of TNBC cells [68]. The circRNA circFUT8 was downregulated and proved to be tumour suppressor in bladder cancer through the miR-570-3p/KLF10 axis [69]. At last but not at least, deregulation of m^6^A modifications in both pri-miRNA and mRNA processing has been discovered in many cancers. The “writers” METTL3 and METTL14 have been found to be highly abundant in some types of cancers, they exert their oncogenic role by promoting translation of targeted mRNAs through m^6^A modification [70]. Furthermore, they can alter pri-miRNA processing by recruiting DGCR8 in m^6^A-dependent manner in HCC [71].

### Epigenetic alterations in TME

Recently, the studies on epigenetics have paid more attention to the TME, especially in the regulation of the immune system during tumorigenesis. The TME is comprised of variable materials including stromal cells, immune cells, extracellular matrix and cytokines, creating a favourable and immune-suppressive niche for tumour cells. The changes of TME in both stromal compartments and immune response during tumorigenesis are accompanied with epigenetic reprogramming, especially the aberrant landscape of noncoding RNAs.

Accumulating evidence indicated that there exist a large number of extracellular vesicles (EVs) secreted by many cell types in TME, including exosomes, microvesicles, ectosomes, large oncosomes, exosome-like vesicles, and apoptotic vesicles [72]. They contain DNA fragments, mRNAs and noncoding RNAs and serve as pivotal communicating messengers between cells in early stages of premetastatic niche formation and are critically associated with EMT and metastatic progress [73]. For instance, the exosomal miR-200b was higher in pancreatic ductal adenocarcinoma (PDAC) and indicated shorter overall survival (OS) [74]. It was reported that Non-small cell lung cancer (NSCLC) cell-derived exsomes were overexpressed miR-619-5p that induced cancer cells growth and metastasis by suppressing RCAN1.4 [75]. Moreover, the miRNAs translocated to paracancerous cells can deliver oncogenic signals to shape beneficial TME. In the pre-metastatic niche of colon-rectal cancer (CRC) cells, the exosomal miR-25-3p was transferred to endothelial cells to promote vascular permeability and angiogenesis [76]. The BRACA1-KO fibroblasts that exposed to CRC-derived EVs underwent phenotypical transformation, which was likely caused by cancer DNA, mRNA and miRNAs in EVs [77]. MiR-409 and miR-154* are found to be expressed in carcinoma-associated fibroblasts (CAFs) which supposed to be silenced after early embryogenesis [78].

It has been widely known that the whole process of tumorigenesis requires an immune-suppressive environment which enables tumour cells to escape from immune surveillance and T cells-regulated anti-cancer killing. Utilizing the inhibitory immune checkpoint pathway to prevent immune system is one of strategies. The most broadly studied checkpoint proteins are engaged in the surface of both T cells and cancer cells, generally serving as receptor and ligands, including CTLA-4/CD80 or CD86, PD-1/PD-L1 or PD-L2, LAG3/MHC-II, TIM3/galectin-9, BTLA/HVEM, TIGIT/CD155 [79, 80]. Induction of those checkpoint proteins can turn T-cells into “off” status, and it is under epigenetic control in cancers. Lower repressive histone marks and DNA methylation marks are usually found at the promoters of checkpoint proteins. It has been reported that PD-1, CTLA-4, TIM-3 and TIGIT were hypomethylated in tumour tissues, compared to the normal tissues, and they also exhibited reduced H3K9me3 and H3K27me3 at their promoters [81, 82]. In addition, ncRNAs are explicitly contribute to epigenetic control in the immune checkpoint that have been summarized in the reference [83].

Except for the role of immune checkpoints in immune suppression, one of the known barriers for deficiency of tumour-infiltrated lymphocytes is the lack of chemokines, including CCL2, CCL4, CXCL8, CXCL9, CXCL10, CXCL12 and CXCL14 etc. These chemokines are supposed to recruit corresponding immune cells into the TME. However, they are epigenetically suppressed in many cancers. For instance, EZH2 was overexpressed to repress the production of T helper 1 (Th1)-type chemokines CXCL9 and CXCL10 via trimethylation at H3K27, thus establishing an immune suppressive TME for ovarian cancer [84]. In osteosarcomas, CXCL12 was epigenetically reduced by DNMT1 and lead to impair cytotoxic T-cell homing to the cancer cells [85].

## Epigenetics therapy in cancer

Epigenetic alterations have fundamental functions in cancer progression characterized by reversibility and susceptibility to external factors. They are emerging as promising targets for cancer therapies. The drugs that target the epigenome, called epi-drugs, have been developed more than 40 years. Until now, they have been tested in clinical trials for cancer treatments and displayed favourable outcomes to some extent. A summary of epi-drugs currently in clinical trials can be found in Table 1.

**Table 1:** Epi-drugs currently in clinical trial

Target	Drug	Therapeutic Strategy	Cancer/Disease	Phase	Reference/NCT no.
DNMT	Azacytidine (Vidaza®)	Monotherapy	MDS, AML	FDA approved	[86]
		Polytherapy (cytarabine)	AML	III	[158]
	Decitabine (Dacogen®)	Monotherapy	MDS, AML	FDA approved	[87]
		Polytherapy (Talacotuzumab)	AML	III	[159]
	Disulfiram	Polytherapy (Chelated Zinc)	Melanoma	II	NCT02101008
	EGCG	Polytherapy (Green tea)	PC	II	NCT00666562
	hydralazine	Polytherapy (magnesium valproate)	Refractory solid tumour	II	[160]
	SGI-110	Monotherapy	HCC	II	NCT01752933
		Polytherapy (Pemetrexed, Cisplatin, Gefitinib)	NSCLC	II	[161]
	6-thioguanine	Polytherapy (Dexamethasone, Cyclophosphamide, Vincristine etc.)	Lymphoma	IV	[162]
	4′-thio-2′-deoxycytidine (TdCyd)	Monotherapy	Solid tumour	I (recruiting)	[163]
	MG98	Monotherapy	Solid tumour	I	[164]
HDAC	Abexinostat (PCI-24781)	Monotherapy	Lymphoma	I & II	[165]
		Polytherapy (Doxorubicin)	Sarcoma, lymphoma	I & II	[166]
	CUDC-101	Monotherapy	Solid tumour	I	[167]
	Belinostat (Beleodaq /PXD101)	Monotherapy	PTCL; HCC, Burkitt lymphoma, DLBCL, thymic carcinoma, MDS	FDA approved;	[98]
				II	[168]
		Polytherapy (Paclitaxel, Carboplatin)	Ovarian cancer, fallopian tube cancer, bladder cancer	I & II	NCT00421889
	Entinostat (SNDX-275)	Polytherapy (Entinostat, Exemestane, Placebo)	Breast cancer	II	[169]
	Givinostat (ITF2357)	Monotherapy	Polycythemia vera	I & II	NCT01901432
HDAC	Mocetinostat (MGCD0103)	Polytherapy (Gemcitabine)	Metastatic leiomyosarcoma	II	NCT02303262
	Panobinostat (LBH-589)	Monotherapy	MM; thyroid carcinoma, RCC, breast cancer, AML	FDA approved;	[99]
				II	[170]
		Polytherapy (Placebo)	Hodgkin’s lymphoma, MM	III	[171]
	Pracinostat (SB939)	Monotherapy	MLD	II	[172]
		Polytherapy (Ruxolitinib)	MLD	II	NCT02267278
	Romidepsin (Depsipeptide/FK228)	Monotherapy	CTCL, PTCL	FDA approved	[101]
		Polytherapy (Alisertib, Pralatrexate, Gemcitabine)	Relapsed PTCL	III	NCT01482962
	Valproic acid (VPA)	Polytherapy (Azacytidine, All-trans retinoic acid)	MDS, AML	II	[173]
	Vorinostat (SAHA)	Monotherapy	CTCL	FDA approved	[97]
		Polytherapy (KW-0761)	AML	III	[174]
BET	I-BET762 (GSK525762/molibresib)	Monotherapy	Neoplasms	II (recruiting)	NCT01943851
		Polytherapy (Abiraterone, Enzalutamide, Prednisone)	Solid tumour	I (recruiting)	NCT03150056
	OTX-015 (MK-8628)	Monotherapy	AML, DLBCL, ALL, MM	I	[175]
	TEN-010 (RO6870810)	Monotherapy	Advanced solid tumors	I (recruiting)	NCT01987362
		Polytherapy (atezolizumab, daratumumab, venetoclax, rituximab)	Advanced MM	I (recruiting)	NCT03292172
	CPI-0610	Polytherapy (Ruxolitinib)	Myelofibrosis, AML, MDS	I & II (recruiting)	NCT02158858
	FT-1101	Polytherapy (Azacitidine)	AML, non-Hodgkin lymphoma	I	NCT02543879
BET	ZEN003694	Polytherapy (Talazoparib)	TNBC	II (recruiting)	NCT03901469
	BMS-986158	Monotherapy	Lymphoma, brain tumour	I (recruiting)	NCT03936465
		Polytherapy (Nivolumab)	Advanced tumour	I & II (recruiting)	NCT02419417
	ABBV-075	Polytherapy (Vnenetolaclx)	Solid tumour and hematologic malignancies	I	NCT02391480
	GS-5829	Polytherapy (Exemestane, Fulvestrant)	Solid tumour, lymphoma	I	NCT02392611
	PLX51107	Polytherapy (Azacitidine, BRD4 inhibitor)	AML, MDS	I (recruiting)	NCT04022785
ncRNA	MesomiR-1 (miR-16)	Monotherapy	NSCLC, malignant pleural mesothelioma	I	[176]
	Miravirsen (miR-122)	Monotherapy	Hepatitis C	II	[177]
	MRX34 (miR-34)	Monotherapy	Primary liver cancer, SCLC, lymphoma, MM, RCC	I (terminated)	NCT01829971
	MRG110 (anti-miR-92)	Polytherapy (Placebo)	Wound healing	I	NCT03603431
	MRG201 (miR-29)	Polytherapy (Placebo)	Fibrous scar tissue formation	I	NCT02603224
	MRG106 (miR-155)	Polytherapy (Cobomarsen, SAHA)	CTCL, Mycosis fungoides	II (recruiting)	NCT03713320
	Patisiran	Monotherapy	Rare polyneuropathy	FDA approved	[136]
		Polytherapy (Vutrisiran)	Amyloidosis	III (recruiting)	NCT03759379
	RG-012 (anti-miR-21)	Monotherapy	Alport syndrome	I	NCT03373786
	TargomiRs (miR-15/17)	Monothearpy	Malignant pleural mesothelioma, NSCLC	I	[176]

### DNMT inhibitors (DNMTIs)

DNMTIs are potent anticancer therapeutics to reverse the DNA hypermethylation status of TSGs. According to the regulatory mechanisms to the nucleotides, DNMTIs can be divided into two classes: cytosine analogue inhibitors and non-nucleotide analogue inhibitors. In general regards, cytosine analogues can incorporate into the DNA or RNA backbone to replace C-5 of cytosine with N-5 and disturb the methylation, as well as induce DNMTs degradation. They include 5-aza-cytidine (azacytidine), 5-aza-2′-deoxycytidine (decitabine), zebularine, SGI-110, fazarabine, pseudois cytidine, etc.

Azacytidine and decitabine are cytosine analogue inhibitors and have been approved by the FDA for the treatment of hematologic malignancies, specifically myelodysplastic syndrome (MDS) and acute myeloid leukaemia [86, 87]. Nowadays, they are widely implicated in different solid tumours. Azacytidine has a large portion incorporated into RNA, while decitabine is only incorporated into DNA. The action of decitabine starts with DNA integration. After that, the formed azacytosine-guanine dinucleotides trap DNMTs with irreversible covalent bindings, thereby exhausting DNMTs and removing the DNA methylation marks on the promoters of TSGs [88]. Furthermore, DNA damage response is triggered along with this process and leads to cell cycle arrest, growth suppression, and apoptosis. As for azacytidine, given to its capacity in the incorporation of RNA, recent studies demonstrated that it can block gene translation via disrupting tRNA-rRNA interactions and inhibit the conversion of deoxyribonucleotides [89]. The anti-tumour activities of these two drugs have been determined in clinical trials at relatively low doses due to their high toxicity caused by high doses.

Apart from azacytidine and decitabine, there are many other cytosine analogues that function in different mechanisms, such as zebularine (ZEB), 6-thioguanine, and 4′-thio-2′-deoxycytidine. ZEB contains a 2-(1H)-pyrimidinone ring that leads to degradation of DNMTs via forming a covalent complex with DNMTs at position 6 of the pyrimidinone ring after DNA incorporation [90]. Distinct from azacytidine and decitabine, which can be deactivated by cytidine deaminase (CD), ZEB is more stable with a long half-life that enables oral administration [91]. Of note, ZEB has a preferential response to tumour cells because of faster DNA incorporation and higher response [92, 93]. Although ZEB alone is not as efficient as azacytidine or decitabine due to the competitive effect of cytidine deaminase, it facilitates preventing re-methylation of the gene after treatment of other DNMTIs and may lower doses of DNMTIs [94]. For example, p16 expression may occur re-silence by DNA methylation after decitabine treatment in bladder cancer cells. However, with the addition of ZEB administration, the demethylation effect could be maintained [84]. Therefore, it also combines with azacytidine and decitabine and displays much safer in various cancer treatments, such as AML and EBV-positive Burkitt’s lymphoma [95].

In a class of non-nucleotide analogue inhibitors, they are small molecules that prevent the binding of DNMTs to the target sequences either by binding to the catalytic site of DNMTs or binding to the CpG-rich sequences. They include hydralazine, EGCG, RG108, MG98, and disulfiram, etc. Those epi-drugs have slightly inhibitory effects to multiple cancer cells compared with those cytosine analogue inhibitors. MG98 is an antisense oligonucleotide that targets 3’UTR of DNMT1 and induces demethylation in vitro and in vivo [96]. The significant inhibition of DNMT1 expression was observed in phase I clinical study. However, no detectable effect was noted in phase II clinical trial in patients who suffered metastatic renal carcinoma.

### HDAC inhibitors (HDACIs)

HDACIs are capable of rectifying the aberrant acetylation status of histones and non-histone proteins in cancers via reactivation of TSGs. Also, cancer cells exhibit a higher sensitivity in response to HDACIs-induced apoptosis. Those features make them become a promising target in cancer therapy. Based on their structure, HDACIs can be divided into four groups: hydroxamic acids, cyclic peptides, aliphatic fatty acids, and benzamides.

The hydroxamic acid HDACIs contain a hydroxamic acid moiety that can bind to the zinc atom, a component in the catalytic sites of HDACs, thus inactivating HDACs. Multiple studies have demonstrated their success in treating both hematologic malignancies and other solid tumours. Currently, three general hydroxamic acid HDACIs have been approved by FDA: (i) Vorinostat (SAHA) which is responsible for cutaneous T-cell lymphoma (CTCL) [97]; (ii) Belinostat (PXD101) which is responsible for peripheral T-cell lymphomas (PTCL) [98]; and (iii) Panobinostat (LBH-589) which is responsible for multiple myeloma [99]. SAHA is a non-selective broad-spectrum HDACIs that inducing acetylation of histones. It has been reported to enhance the expression of p21 by inducing acetylated histone H3 and H4 in bladder carcinoma and endometrial stromal sarcomas [100]. Also, there are other hydroxamic acid HDACIs similarly displaying inhibitory effects on HDACs either selectively or generally, including resminostat, givinostat, pracinostat, abexinostat, and quisinostat, etc. They have been implicated in phase I or II clinical trials for multiple cancers.

Romidepsin (FK2280) is a member of cyclic peptide HDACIs and has received approval of the FDA in 2009 and 2011 for the treatment of CTCL and PTCL respectively [101]. It undergoes reduction by glutathione and releases a zinc-binding thiol within cells. With this thiol, FK2280 interacts more explicitly with class I/II HDACs, leading to reactive target genes [102, 103].

Valproic acid (VPA) is an example of aliphatic fatty acid HDACIs and selectively targets class I/II HDACs. It was originally developed for the treatment of epilepsy, and its application was then extended to anti-tumour treatment due to its ability in suppressing the proliferation and stimulating the differentiation of cancer cells. VPA can increase histone H3 and H4 acetylation and cause demethylation of target genes, especially in nondividing cells. Some of the genes activated by VPA are associated with cancer metastasis (e.g., MMPs) or tumour-specific antigens (e.g., MAGEB2) [104]. Moreover, VPA has a property of low toxicity, well tolerance, and stability, which makes it a promising epi-drug. Phenylbutyrate, AR-42, and pivanex (AN-9) are other members of short-chain fatty acid HDACIs.

Benzamide derivative was firstly reported by Suzuki et al. in the 1990s and displaying significant HDACs inhibition [105]. Entinostat (MS-275) and tacedinaline (CI-994) are very active HDACIs of the benzamide group. They possess a 2′-aminophenyl group that binds to the specific site of class I HDACs and display inhibitory effect on HDACs. The treatment of MS-275 could induce expression of multiple genes and increased the overall acetylation status of histones in vitro and in vivo [106]. It was also well-tolerated for patients with lymphoid malignancies and solid tumours in phase I and II clinical trials. Similarly, CI-994 has been tested in phase II clinical trials and could be used alone or in combination with other chemotherapeutic drugs to treat solid tumours in patients [107, 108].

### Bromodomain and extra terminal inhibitors (BETIs)

The BET is a family of proteins that contain two N-terminal tandem bromodomains and a C-terminal extra terminal motif, including BRD2, BRD3, BRD4, and BRDT. They form a complex in association with HDACs and other proteins to stimulate transcriptional activity. BRD4 is the most characterized BETs which is exceptionally involved in transcriptional regulation and cancer progression due to its capacity of assembling on both hyper-acetylated gene promoters and “super-enhancers” to promote RNA-polII-mediated transcriptional initiation and elongation [109, 110]. Several oncogenes have been described as the effectors of BRD4, including c-Myc, FOSL1, RUNX2, BCL-2, and c-KIT [111–114]. The efficacy of BETIs is based on the disruption of BETs-acetylated histones interaction. So far, several BETIs have encouraging clinical outcomes with tolerable toxicity and potent efficacy, including thienodiazepine JQ1, I-BET762 (GSK525762), I-BET151 (GSK1210151A), GS-5829, CPI-0610, TEN-010, OTX-015, and ZEN003694.

JQ1 has been reported to block the interactions of BRD2/3/4 and acetylated histones selectively. One of the target genes c-Myc is subject to downregulation by JQ1 in many different cancers [115]. Furthermore, it exhibits a drastically anti-tumour activity even in castration-resistant cells by disrupting BRD4-mediated androgen receptor (AR) recruitment and transactivation. Recent studies have reported that JQ1 also increased cytotoxic T-cell response by increasing PD-L1 expression [116]. A new oral derivative of JQ1, OTX015, has been synthesized to function as an inhibitor of BRD2/3/4 [117]. Administration of OTX015 could diminish the phenotype of CSCs, providing a compelling strategy in the most aggressive PC [118].

### Histone methyltransferases/demethylases inhibitors

EZH2 is a histone methyltransferase and is responsible for the catalytic activity of PRC2. EZH2/PRC2 methylates H3K27 and leads to transcriptional silence of target genes in multiple subtypes of cancers, including ovarian cancer, breast cancer, PC, T-cell ALL and non-Hodgkin lymphoma [119–128]. Small molecule EZH2 inhibitors, such as EPZ-6438 (tazemetostat), GSK2816126, and CPI-1205, have been evaluated in clinical trials and showed antineoplastic effects in both hematologic malignancies and various solid tumours. EPZ-6438 is an orally bioavailable EZH2 inhibitor by competing with SAM, which is a cofactor of EZH2 [128]. With encouraging phase I and II clinical trials, EPZ-6438 has been granted as a Fast Track designation for diffuse large B cell lymphoma (DLBCL) and follicular lymphoma (FL), as well as Orphan Drug designation for malignant rhabdoid tumours by FDA [129].

Another group of epigenetic drug precursor is histone demethylase inhibitor, including inhibitors of LSD1 and Jumonji (JmjC). The effect of LSD1 inhibitors is based on the impairment of the H3K4 demethylation [130]. LSD1 inhibitors have been grouped into four classes: (i) reversible poly- or monopeptide inhibitors, (ii) irreversible derivatives of monoamine oxidase inhibitors, (iii) rationally designed fusions of active molecules, and (iv) novel compounds not known to inhibit monoamine oxidase (MAOI). For example, the highly selective LSD1 inhibitors, such as namoline, HCI-2509, NCL-1, have been identified to be reversible inhibitors that have the robust capability of impairing H3K4 demethylation. The treatment of these LSD1 inhibitors can lead to androgen-independent growth arrest without apparent adverse effects in castration-resistant PC cells both in vitro and in vivo [131–133]. The inhibitors of JmjC, which include hydroxamate derivatives, pyridine dicarboxylates, N-oxalyl amino acid derivatives, and agents that interfere with metal binding, have been designed by targeting the 2-OG of the JmjC family, which is a key component for demethylation of methylated lysine [134, 135].

### NcRNAs

Increasing studies have shown that miRNAs become biomarkers of multiple cancers as their abnormal levels may be considered to indicate the stage of pathology and prognosis. The applications of miRNAs analogue or anti-miRNAs have shown compelling outcomes in-vitro and in-vivo cancer studies, suggesting the miRNA-based drugs are emerging as a novel strategy for cancer therapy. Nowadays, the first small-interfering RNA (siRNA), patisiran, has got approval from the FDA in 2018 for the treatment of rare polyneuropathy via targeting and degrading the mRNA transcription of transthyretin [136]. Many other miRNA-based drugs are under clinical trials and may be translated into FDA-approved drugs in the future, including liposome-formulated miR-34a mimic (MRX34), MRG110, MRG-201, MRG-106, RG-012, RGLS5579, and TargomiRs (miR-15/17 consensus sequences). They either function as miRNA mimics or miRNA antagonists in the treatments of different cancers. For example, the expression of tumour suppressor miRNA-34a is usually relatively low in various cancers, such as PC, NSCLC, and ALL, and it has been identified as a tumour suppressor with multiple targets, including CD44, PD-L1, ZEB1, and BCL-2. MRX34 has been developed as a novel strategy to increase the miR-34a level for cancer therapy and has been launched a phase II clinical trial [137]. In terms of higher expression of onco-miRNAs in specific malignancies, the antagomiR-mediated inhibition of specific miRNAs is highly recommended in clinical trials. A latest study has shown that antagomiR-214 decreased disease severity in CTCL, which provides another angle of view for CTCL therapy [138]. Although JQ1 could also downregulate miR-214, indicating the upregulation of miR-214 was caused by aberrant histone acetylation [138].

Numerous lncRNAs are subsequently identified and found to be aberrantly expressed in various tumours. Due to the multifaceted regulations and intricate mechanisms of lncRNAs, few of them have been already implicated as drugs for clinical therapies. However, they are capable of functioning as indicative of the severity of specific cancers as they are supposedly stable in body fluid. For example, the higher lncRNA PCA3 in urine may correspond to the severity of prostate cancer [139]. LncRNA MALAT1, NEAT1, UCA1, and ANRIL can seem as biomarkers for metastatic lung cancers [140]. Similar to the miRNA-mediated strategies, antisense-mediated lncRNA targeting is also shown to be a promising tool for some cancer therapies [141]. So far, there are still many hurdles regarding the clinical application of ncRNAs; introducing those ncRNAs with efficient delivery systems is always a potential strategy in cancer therapy.

### Epi-drugs combined therapy

The administration of multiple epi-drugs themselves or in combination with chemotherapy and immunotherapy is becoming a novel approach due to its augmentation of anti-tumoral effects and overcoming drug resistance. The impact of multiple epi-drugs treatments is based on the synergistic actions of epi-drugs. For example, SAHA and panobinostat co-treatment displayed robust anti-cancer activity in colon adenocarcinoma and leukaemia via inducing immunogenic cell death (ICD) [142]. The treatment of SAHA with panobinostat is reported to epigenetically silence the AR gene and repress transcription of the AR targeted genes PSA and tMPRSS2 [143]. The combination of JQ1 or EZH2 inhibitors with panobinostat presents synergistic effects in vitro and in vivo [144, 145].

So far, chemotherapy is still a traditional method for advanced cancers that lack the opportunity for surgical excision. The emergence of chemoresistance becomes a great hindrance to cancer therapy. One of the reasons is that some chemotherapy agents can bring into aberrant epigenetic changes after treatment. For example, cisplatin treatment in ovarian cancer can induce hypermethylation of multiple genes (e.g., MLH1, MEST, MDK) and lead to the acquired resistance phenotype [146]. The addition of decitabine can abate and even reverse the resistance to cisplatin via re-activating those epigenetic-silenced genes, suggesting that combining epi-drugs with other chemotherapeutic agents can not only remarkably promote potent suppression of tumorigenesis, but also re-sensitize tumour cells to radiotherapy and chemotherapy. The studies of HCC have found HDAC3 and HDAC5 mRNAs were upregulated. Inhibiting those HDACs with LBH-589 could increase acetylation of histone H3 and HSP90 and upregulate CDH1 [147]. The SHELTER studies also investigated that the combination of sorafenib (an FDA approved systemic drug) and resminostat, which is an oral pan-HDACi with predominant activity against HDAC1, 2, and 3, showed a compelling outcome with a 2-fold increase of overall survival (OS) in a second-line setting of advanced HCC, compared to monotherapy of resminostat [148].

Immune checkpoint inhibitors (ICIs) have been considered as a great breakthrough for the treatment of malignant diseases. PD-1/PD-L1 and CTLA-4 inhibitors are the most promising ICIs that some of them have been approved for certain malignancies therapies. However, the clinical benefit of ICIs is obstructed by the lack of surface markers for antigen presentation, impaired T cell response, re-educated TME, etc. To some extent, those hinders can be overcome by epigenetic reprogramming. Recent studies have provided evidence that the efficacy of PD-1 antibodies can be potentiated by using the DNMTIs. In breast cancer, the major histocompatibility class-I (MHC-I)-encoding genes were methylated and sustained in silence expression status. The treatment with guadecitabine, one of DNMTIs, could enhance MHC-I expression and increase CD8+ T cell infiltration in TME [149]. Those reactions could further potentiate subsequent responses to PD-1 antibodies. Another example is the effect of decitabine in sensitizing CD8+ T cells to PD-L1 antibodies via disrupting the DNMT3A-mediated methylation in exhausted T cells [150]. The administration of belinostat was reported to induce CTLA-4 inhibition, which was responsible for M1-phenotypic tumour-associated macrophages (TAMs), and decrease splenic regulatory T cells (Tregs) [151]. Many other epi-drugs (e.g. JQ1, LSD1 inhibitors, and EZH2 inhibitor) in combination with anti-PD1 therapy, have been revealed that they can increase anti-tumour immune response via enhancing T-cell persistence in different cancers, such as lung cancer, TNBC, and lymphoma, etc. [116, 152]

## Discussion

Notwithstanding, compelling evidence has described that implying epi-drugs alone or in combination with other drugs in clinical trials, can improve the anti-tumour efficacy. However the accompanying problems may not be underestimated. Firstly, a considerable number of epigenetic compounds are still ongoing in laboratory investigation. The major challenge for those compounds would be how to translate the efficacy in vitro with nanomolar-scale concentrations into well-tolerated and efficient clinical use. MG98 has been found to efficiently reactivate silenced TSGs via downregulation of DNMT1 in several cancer cell lines at concentrations of 25–76 nM and presented inhibitory effect on proliferation. However, it did not achieve significant response in clinical trials [153].

The off-target of epi-drugs is another problematic issue. As epigenetic regulation is multifaceted, dynamic, and interdependent, the mechanism behind is not clear. Currently, some commonly used epi-drugs, such as VPA, have been known to generate unwanted epigenetic modifications [154, 155]. A well-established safety profile of those epi-drugs are needed to make ease for their applicability in clinical therapy. Meanwhile, the acquired resistance to some of the epi-drugs is becoming a hurdle. The BETIs resistant AML cells displayed PRC2 suppression, which may recover the BETI-targeted c-Myc expression [156]. The hyperphosphorylation of BRD4 is also promoting BETIs resistance in triple-negative breast cancer (TNBC) [157].

## Conclusion and future perspectives

Epigenetics represents a series of dynamic alterations which is independent of genetic changes. They are critically involved in the onset and development of tumorigenesis by regulating the on and off states of oncogenes and TSGs, as well as TME reengineering. Remarkably, epigenetic modifications feature properties of heritable and reversible regulations, making them a promising target for cancer therapy. Current epi-drugs have been used in different cancer types either in a single treatment or a combined treatment with other anti-cancer agents, showing compelling outcomes in some extent. However, the subsequent shortcomings are needed to be considered.

Since human cancer features properties of heterogeneity and plasticity, the request for a precise and effective personalized therapy using epi-drugs is being brought into concern. The standard anti-cancer therapy for general cancer patients has received very limited prognosis due to individual differences. With the advent of high throughput epigenome mapping technologies, the genome and epigenome map of a specific cell population from the patient are available for drug sensitivity testing and drug screening. In this way, the treatments can be optimized for each patient while having much efficiency and less off-target effects.

## Data Availability

Not applicable.

## Abbreviations

5-caC: 5-carboxylcytosine
5-fC: 5-formylcytosine
5-hmC: 5-hydroxymethylcytosine
m5C: 5-methylcytosine
AR: Androgen receptor
BETIs: Bromodomain and Extra Terminal inhibitors
CAFs: Carcinoma-associated fibroblasts
CGIs: CpG islands
CDKN2A: Cyclin-dependent kinases inhibitor 2A
CD: Cytidine deaminase
CTLA-4: Cytotoxic T-lymphocyte associated protein 4
DAPK: Death-associated protein kinase
DLBCL: Diffuse large B cell lymphoma
DNMTs: DNA methyltransferases
DNMTIs: DNMT inhibitors
EZH2: Enhancer of zeste 2
EMT: Epithelial-mesenchymal transition
eIF3: Eukaryotic initiation factor 3
GSTP1: Glutathione S-transferase pi
H3K27me3: H3K27 trimethylation
HDACIs: HDAC inhibitors
HCC: Hepatocellular carcinoma
HNRNP: Heterogenous nuclear ribonucleoprotein
H2A: Histone 2A
H2B: Histone 2B
H3: Histone 3
H4: Histone 4
HATs: Histone acetyltransferases
HDACs: Histone deacetylases
HDMTs: Histone demethylases
HMTs: Histone methyltransferases
HIF-1α: Hypoxia-induced factors 1α
ICIs: Immune checkpoint inhibitors
ICD: Immunogenic cell death
IGF2BP: Insulin-like growth factor 2 mRNA binding proteins
lncRNAs: Long ncRNAs
LAG-3 with galectin-9: Lymphocyte activating 3
H3K4/79: Lysine 4/79 of histone H3
MHC-I: Major histocompatibility class-I
MBDs: Methyl-binding domain proteins
METTL3: Methyltransferase-like 3
MDS: Myelodysplastic syndrome
m^6^A: N^6^-methyladenosine methylation
ncRNAs: Non-coding RNAs
OS: Overall survival
PTCL: Peripheral T-cell lymphomas
PTM: Post-translational modifications
PD-1: Programmed death-ligand 1
PC: Prostate cancer
Tregs: Regulatory T cells
RMB15/15B: RNA binding motif protein
SAM: S-adenosylmethionine
snRNAs: Small ncRNAs
siRNA: Sall-interfering RNA
Th1: T helper 1
TIM-3: T-cell immunoglobulin mucin 3
CTCL: T-cell lymphoma
TIMPS: Tissue inhibitors of metalloproteinases
TFs: Transcriptional factors
TNBC: Triple-negative breast cancer
TME: Tumour microenvironment
TSGs: Tumour suppressors genes
TAMs: Tumour-associated macrophages
VPA: Valproic acid
WTAP: Wilms tumour 1-associated protein
YTH: YT521-B homology
ZEB: Zebularine
ZC3H13: Zinc finger CCCH-type containing 13
